# Laparoscopic ureteral anastomosis to a bladder diverticulum for the treatment of postoperative ureteral fistula: a case report

**DOI:** 10.3389/fmed.2026.1876215

**Published:** 2026-08-26

**Authors:** Jiansheng Xiao, Guohao Wu, Ping Li, Handa Zheng, Zhihui Chen, Feng Luo, Huilan Luo, Caiyong Lai, Dongming Ye

**Affiliations:** 1 Department of Urology, The Sixth Affiliated Hospital of Jinan University, Dongguan Eastern Central Hospital, Dongguan, China; 2 Department of Urology, Miyun Hospital, Peking University First Hospital, Beijing, China

**Keywords:** bladder diverticulum, case report, treatment, ureteral fistula, ureteral reimplantation

## Abstract

**Objective:**

To investigate the feasibility and potential indications of laparoscopic ureteral anastomosis to a bladder diverticulum for the treatment of ureteral fistula in the setting of complex reoperative pelvic surgery.

**Case presentation:**

A 75-year-old man with a 7-year history of progressive dysuria underwent transabdominal laparoscopic bladder diverticulectomy and cystolithotomy combined with transurethral holmium laser enucleation of the prostate for benign prostatic hyperplasia–related bladder outlet obstruction, bladder stones, and bladder diverticula. Twelve days after surgery, the patient developed increased pelvic drainage output. Elevated drainage-fluid creatinine levels and computed tomography urography showing contrast extravasation from the left ureter with hydronephrosis confirmed a left ureteral fistula. During reoperation, severe adhesions around the left ureter and bladder precluded conventional reconstruction. A bladder diverticulum adjacent to the distal ureter was therefore used as an alternative implantation site, and laparoscopic ureteral reimplantation into the diverticulum was performed. At 4 months of follow-up, no progression of hydronephrosis, recurrent urinary leakage, or urinary tract infection was observed.

**Conclusion:**

In selected patients with postoperative ureteral fistula, severe perivesical adhesions, and limited feasibility of conventional reconstruction during complex reoperative surgery, ureteral anastomosis to a bladder diverticulum may serve as an individualized salvage option for urinary tract reconstruction.

## 1 Introduction

Ureteral fistula is an uncommon but clinically significant complication that most often results from iatrogenic ureteral injury during pelvic or abdominal surgery (1–3). If not promptly recognized and managed, it may lead to urinary extravasation, abdominal or pelvic infection, hydronephrosis, and subsequent impairment of renal function on the affected side (4, 5). Because ureteral injury may be missed intraoperatively, the diagnosis is often established postoperatively based on clinical manifestations such as increased drainage output, elevated creatinine levels in the drainage fluid, flank pain, fever, or vaginal urinary leakage, together with imaging evidence of hydronephrosis or urinary extravasation (6, 7). Early recognition, accurate localization, and timely intervention are therefore essential for controlling infection, preserving renal function, and improving clinical outcomes in patients with suspected postoperative urinary leakage (8).

The management of ureteral fistula should be individualized according to the site and timing of injury, extent of urinary leakage, severity of local infection or inflammation, length of the ureteral defect, bladder condition, and renal function of the affected side (9–11). Treatment options range from ureteral stent placement, percutaneous nephrostomy, and adequate drainage to definitive reconstruction, including ureteroureterostomy, ureteral reimplantation, and, when required, psoas hitch or Boari flap reconstruction. For distal ureteral injury or fistula formation, ureteral reimplantation is a key reconstructive option (12). Its surgical principles include excision of devitalized or fibrotic tissue, preservation of ureteral blood supply, avoidance of ureteral kinking or torsion, creation of a tension-free anastomosis, and adequate postoperative drainage (13).

However, these standard reconstructive strategies may be difficult to apply in the setting of early postoperative reoperation or complex reoperative pelvic surgery. Local inflammatory edema, dense adhesions, and altered anatomy can make mobilization of the distal ureter and bladder technically challenging, thereby limiting conventional ureteral reimplantation, psoas hitch, or Boari flap reconstruction. In selected cases, a bladder diverticulum that communicates clearly with the bladder lumen and is anatomically close to the distal ureter may provide a potential alternative anastomotic site; however, this approach has rarely been described. Herein, we report a case of postoperative left ureteral fistula treated with laparoscopic ureteral anastomosis to a bladder diverticulum in the setting of complex reoperative surgery and severe perivesical adhesions. We further discuss the diagnostic approach, surgical decision-making, technical considerations, and potential indications for this individualized salvage reconstructive strategy.

## 2 Case presentation

A 75-year-old man was admitted to our hospital with a 7-year history of progressive dysuria, which had worsened over the preceding 20 days. Preoperative magnetic resonance imaging revealed significant benign prostatic hyperplasia (prostate volume approximately 115.3 ml), multiple bladder stones, and multiple bladder diverticula accompanied by diverticular stones. Given the long-standing history of bladder outlet obstruction and the associated complications, including bladder stones and diverticula, the diverticula were considered acquired lesions secondary to chronic bladder outlet obstruction. The patient subsequently underwent transabdominal laparoscopic bladder diverticulectomy and cystolithotomy, combined with transurethral holmium laser enucleation of the prostate, to relieve bladder outlet obstruction and address the associated bladder complications.

**Figure 1:**
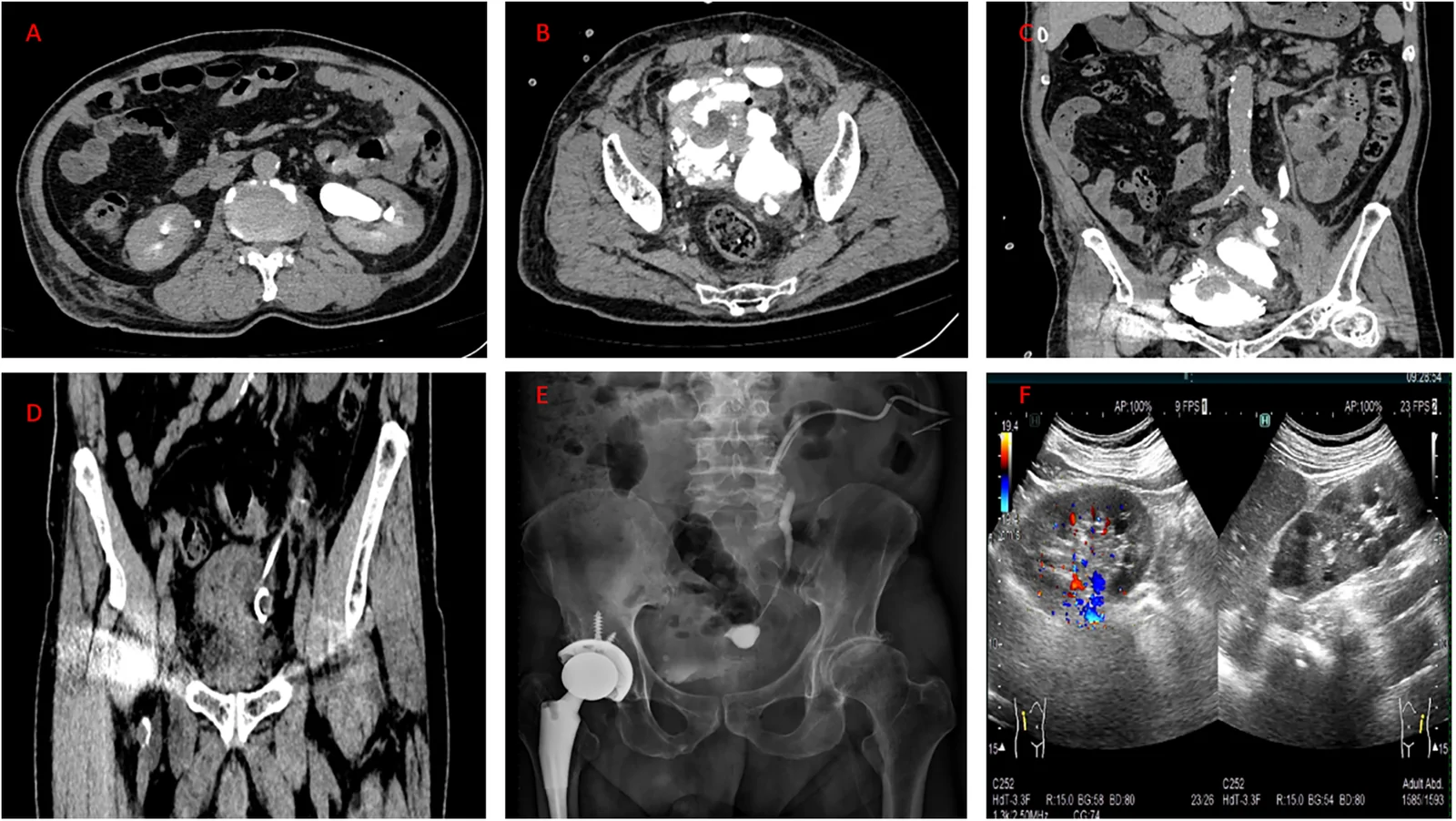
Imaging findings before surgery and during postoperative follow-up. **(A)** Preoperative axial computed tomography urography (CTU) urography image showing dilation and hydronephrosis of the left renal pelvis and proximal ureter. **(B)** Preoperative axial pelvic CTU urography image showing contrast extravasation around the operative site. **(C)** Preoperative coronal CTU urography reconstruction showing left ureteral dilation with contrast extravasation at the operative site. **(D)** Postoperative coronal lower abdominal and pelvic CT image showing the distal end of the double-J stent within the bladder. **(E)** Antegrade urography performed 6 weeks after surgery showing a patent left ureteral anastomosis to the bladder diverticulum, with contrast passage into the bladder lumen and no obvious leakage. **(F)** Urinary ultrasonography performed 4 months after surgery showing no obvious hydronephrosis of the left kidney or left ureter.

Twelve days after the procedure, the patient developed severe tenesmus and a significant increase in pelvic drainage output. The creatinine concentration of the drainage fluid was 2,838.97 μmol/L, which was markedly higher than the serum creatinine level. Computed tomography urography demonstrated contrast extravasation at the operative site, with left hydronephrosis and ureteral dilatation (Figures 1A–C). Laboratory tests showed a serum creatinine level of 101.00 μmol/L, blood urea nitrogen level of 4.95 mmol/L, normal white blood cell count, and a hemoglobin level of approximately 132 g/L, with no other notable abnormalities. Based on the elevated drainage fluid creatinine level, urinary extravasation on computed tomography urography, and left upper urinary tract dilatation, left ureteral fistula was diagnosed. Preoperatively, the patient was classified as American Society of Anesthesiologists physical status II and underwent surgery under general anesthesia. Perioperative antibiotic prophylaxis was administered with cefoperazone 1.5 g intravenously every 12 h. Laparoscopic repair with planned left ureteral reimplantation was initially considered.

### 2.1 Surgical procedure

**Figure 2:**
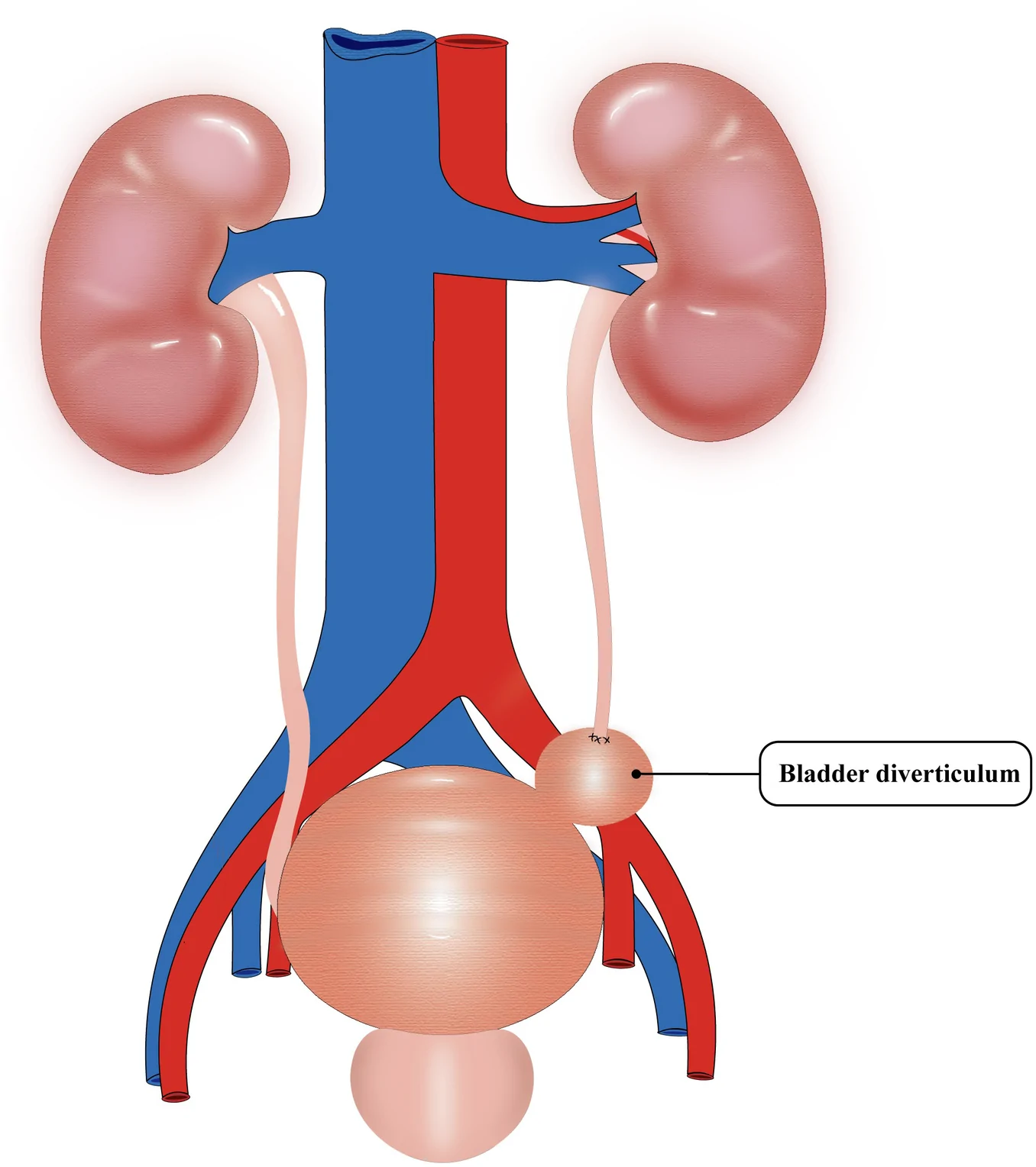
Schematic diagram illustrating the bladder diverticulum used for laparoscopic ureteral anastomosis in the treatment of postoperative ureteral fistula.

Under general anesthesia, the patient was placed in the supine Trendelenburg position. Trocars were inserted through the previous five laparoscopic port sites, and pneumoperitoneum was established. Intraoperatively, localized pelvic edema and dense adhesions involving the left sigmoid colon, left ureter, and bladder were observed, resulting in poorly defined anatomical planes. After meticulous adhesiolysis, the left ureter was identified near the left iliac vessels and dissected distally along its course. A fistula arising from an injury to the distal left ureter was identified. The affected ureteral segment was transected and trimmed, and approximately 1 cm of devitalized ureteral tissue was excised. A 6-F double-J ureteral stent (Bard Medical, Shanghai, China) was prepared for subsequent reconstruction. Conventional left ureteral reimplantation was initially planned. However, severe perivesical adhesions and limited bladder mobility made extensive bladder mobilization or Boari flap creation technically risky; therefore, bladder flap reconstruction was not performed. After the bladder was filled intraoperatively, a left-sided bladder diverticulum was identified. The diverticulum was confirmed to communicate freely with the bladder lumen and was located adjacent to the distal left ureter. To reduce anastomotic tension, the bladder was mobilized superolaterally to the left and fixed to the region of the left psoas tendon. An incision was then made at an appropriate site on the diverticular wall. The distal end of the double-J stent was advanced into the bladder lumen, and the transected end of the left ureter was anastomosed to the bladder diverticulum using 5-0 absorbable sutures. During the anastomosis, particular attention was paid to mucosa-to-mucosa approximation and avoidance of ureteral twisting or tension. At the end of the procedure, the bladder was filled through the urethral catheter, and no obvious leakage was observed. An abdominal drain was placed before closure (Figure 2).

### 2.2 Postoperative management and follow-up

Postoperatively, the urethral catheter and pelvic drain were left in place, and the patient received anti-infective therapy, fluid replacement, and supportive care. The creatinine level in the drainage fluid decreased markedly to 65.17 μmol/L, while serum creatinine and blood urea nitrogen levels were 65.87 μmol/L and 5.32 mmol/L, respectively. The white blood cell count remained normal, suggesting control of urinary extravasation and stable renal function. The pelvic drain was removed on postoperative day 3, and the patient recovered uneventfully. Follow-up lower abdominal and pelvic computed tomography showed that the distal end of the double-J stent was positioned within the bladder (Figure 1D). Six weeks after surgery, the patient returned for left ureteral stent removal and underwent antegrade urography. Contrast medium passed through the left ureter into the bladder diverticulum and then into the bladder lumen, demonstrating a patent anastomosis without obvious leakage (Figure 1E). Four months after surgery, urinary ultrasonography showed no obvious hydronephrosis of the left kidney or ureter (Figure 1F). During follow-up, the patient remained free of flank pain, fever, and recurrent urinary leakage, indicating a favorable short-term outcome. At the latest follow-up, the patient reported significant improvement in urinary symptoms and expressed satisfaction with the treatment outcome. Compared with the preoperative condition, urinary function and daily activities were substantially improved. The patient did not experience recurrent urinary leakage, flank pain, fever, or other symptoms suggestive of urinary tract infection during follow-up. The timeline of the patient's clinical course and management is summarized in Table 1.

**Table 1:** Timeline of clinical events.

Time point	Clinical event
Index surgery (Day 1)	Transabdominal laparoscopic bladder diverticulectomy and cystolithotomy combined with transurethral holmium laser enucleation of the prostate were performed for benign prostatic hyperplasia-related bladder outlet obstruction, bladder stones, and diverticula.
Presentation (Postoperative day 12)	The patient developed severe tenesmus and increased pelvic drainage output.
Diagnosis (Postoperative day 12)	Elevated drainage fluid creatinine and CT urography confirmed left ureteral fistula with urinary extravasation.
Reoperation (Postoperative day 12)	Laparoscopic ureteral reimplantation into a bladder diverticulum was performed due to severe pelvic adhesions and limited feasibility of conventional reconstruction.
Stent removal (Postoperative week 6)	Double-J stent was removed, and antegrade urography showed a patent anastomosis without leakage.
Follow-up (Postoperative month 4)	No progressive hydronephrosis or recurrent urinary leakage was observed; urinary symptoms and quality of life improved.

## 3 Discussion

Ureteral fistulas most commonly arise from ureteral injury, ischemic necrosis, local infection, or impaired anastomotic healing after pelvic or abdominal surgery (14, 15). Because ureteral injury may be missed intraoperatively, postoperative manifestations are often non-specific. Increased drainage output, elevated creatinine levels in the drainage fluid, flank pain, fever, abdominal or pelvic fluid accumulation, and hydronephrosis should raise suspicion for urinary extravasation (6). In the present case, the patient developed increased drainage output after laparoscopic bladder diverticulectomy, cystolithotomy, and transurethral holmium laser enucleation of the prostate, with a markedly elevated creatinine level in the drainage fluid. Computed tomography urography demonstrated contrast extravasation at the operative site, together with left hydronephrosis and ureteral dilatation. These clinical and imaging findings established the diagnosis of left ureteral fistula. This case highlights that, in patients with unexplained increased drainage output after pelvic surgery, timely measurement of drainage fluid creatinine combined with imaging evaluation, particularly computed tomography urography, may facilitate early identification of the source of urinary leakage and the site of ureteral injury.

The management of ureteral fistula should be individualized according to the location and timing of injury, extent of urinary leakage, infection status, renal function of the affected side, length of the ureteral defect, and local tissue conditions (1, 16). For early, mild, or incomplete injuries, conservative or minimally invasive approaches, including ureteral stent placement, percutaneous nephrostomy, adequate drainage, and anti-infective therapy, may achieve spontaneous healing in selected patients. However, distal ureteral injuries associated with persistent urinary leakage, ureteral defects, or unfavorable tissue conditions often require definitive reconstruction. The fundamental principles of ureteral reconstruction include preservation of ureteral blood supply, removal of nonviable tissue, avoidance of anastomotic tension and ureteral kinking, and establishment of durable, low-pressure urinary drainage while minimizing postoperative complications such as leakage, stricture, and reflux (17).

When direct ureteroneocystostomy is not feasible because of insufficient ureteral length or excessive tension, several reconstructive techniques have been developed. Adequate bladder mobilization, psoas hitch, and Boari flap reconstruction are commonly used methods for bridging distal ureteral defects (18, 19). However, these techniques require sufficient bladder capacity, adequate bladder mobility, and favorable local tissue conditions, which may be difficult to achieve in patients with severe pelvic fibrosis, dense adhesions, or previous pelvic surgery. For more extensive or complex ureteral defects, alternative approaches have also been described. Appendiceal interposition and Yang-Monti ileal ureter substitution may provide reconstructive options in selected patients, particularly when a suitable bowel segment is available (20). Nevertheless, these procedures require bowel manipulation and may be associated with mucus production, metabolic abnormalities, and bowel-related complications. Ileal ureter substitution is generally reserved for long-segment ureteral defects but involves greater surgical complexity and potential long-term metabolic consequences. Renal autotransplantation can be considered for extremely complex ureteral injuries when conventional reconstruction is not possible; however, it requires vascular reconstruction and represents a highly invasive procedure (21). More recently, buccal mucosa graft ureteroplasty has emerged as a tissue-preserving option, particularly for ureteral strictures, although its role in acute postoperative ureteral fistula with severe pelvic adhesions remains to be established.

In complex reoperative surgery, however, the choice of reconstruction is often determined not only by the theoretical advantages of each technique but also by the actual intraoperative anatomical conditions. In the present case, reoperation was performed shortly after the initial surgery, when marked pelvic edema, dense adhesions around the left ureter and bladder, and poorly defined anatomical planes were encountered. Although the left ureter was successfully identified and mobilized after meticulous adhesiolysis, extensive perivesical adhesions significantly limited bladder mobility. Under these circumstances, aggressive bladder mobilization or Boari flap creation could have increased the risks of additional bladder injury, bleeding, urinary leakage, and injury to adjacent structures (22). Therefore, conventional bladder-based reconstruction was considered technically challenging in this specific setting.

The distinctive feature of this case was the intraoperative identification of a left-sided bladder diverticulum adjacent to the distal ureteral segment. Importantly, ureteral implantation into the bladder diverticulum was not a preplanned alternative intended to replace conventional reconstructive procedures. Rather, it was an individualized decision based on intraoperative findings. After mobilization and fixation of the bladder, the diverticulum was confirmed to communicate with the bladder lumen and provided an accessible implantation site that allowed relatively tension-free ureteral anastomosis. Therefore, the transected end of the left ureter was reimplanted into the diverticular wall, avoiding further extensive dissection of the severely adherent bladder and reducing the potential risk associated with forced bladder reconstruction. The relatively high location of the diverticulum and unobstructed communication with the bladder lumen may have contributed to effective drainage in the short-term follow-up.

It should be emphasized that ureteral anastomosis to a bladder diverticulum is not a standard form of ureteral reimplantation and should not be regarded as a substitute for established reconstructive techniques. The rationale for this approach was not that the diverticular wall was superior to normal bladder tissue, but that, in this highly selected case, the diverticulum represented an anatomically accessible and technically feasible option for restoring urinary continuity when conventional reconstruction was limited. Therefore, the value of this report lies in demonstrating the importance of individualized intraoperative decision-making in complex ureteral reconstruction rather than proposing a new standard surgical procedure.

Postoperative biochemical and imaging assessments are essential for evaluating the success of this uncommon reconstructive approach. In the present case, the creatinine level in the drainage fluid decreased markedly after surgery, indicating effective control of urinary extravasation. At 6 weeks postoperatively, after removal of the ureteral stent, antegrade urography demonstrated smooth passage of contrast medium through the left ureter into the bladder diverticulum and subsequently into the bladder lumen, with a patent anastomosis and no obvious contrast leakage. At 4 months postoperatively, follow-up urinary ultrasonography showed no obvious progression of left hydronephrosis or ureteral dilatation. The patient remained free of flank pain, fever, and recurrent urinary leakage during follow-up. These findings suggest that ureteral reimplantation into a bladder diverticulum achieved satisfactory short-term urinary drainage in this carefully selected patient.

This case report has several limitations. First, it represents a single case, and the level of evidence is therefore limited. The general safety, reproducibility, and long-term efficacy of this approach cannot be established based on this report alone. Second, the follow-up duration remains relatively short. Since this reconstruction does not incorporate the traditional anti-reflux mechanism of standard ureteroneocystostomy, potential long-term outcomes, including vesicoureteral reflux, recurrent urinary tract infection, anastomotic stricture, impaired diverticular emptying, stone formation, and changes in renal function, require further observation. Therefore, ureteral anastomosis to a bladder diverticulum should only be considered as an individualized salvage option in highly selected patients when conventional reconstruction is technically limited and appropriate anatomical conditions are present. Furthermore, because this approach does not reproduce the anti-reflux mechanism of conventional ureteral reimplantation, careful long-term follow-up is essential to evaluate potential reflux-related complications.

## 4 Conclusion

For patients with postoperative ureteral fistula in whom severe perivesical adhesions limit conventional ureteral reimplantation or bladder flap reconstruction during complex reoperative surgery, ureteral anastomosis to a bladder diverticulum may represent a feasible individualized salvage option for urinary tract reconstruction. Its application requires comprehensive assessment of diverticular communication with the bladder lumen, local tissue quality, and anastomotic tension.

## Data Availability

The original contributions presented in the study are included in the article/Supplementary Material, further inquiries can be directed to the corresponding authors.
